# Some Simple Food Tests

**Published:** 1898-10

**Authors:** Edward A. Martin

**Affiliations:** New York


### SOME SIMPLE FOOD TESTS.

By Dr. Edward A. Martin, of New York.

Dr. Edward A. Martin, Chief of the Food Inspection Division of the Health Department, New York city, said recently that the heads of families, not only in the great metropolis, but throughout the country generally, have within easy reach the means of self-protection against deleterious food products, which, if put in universal practice, would soon make food adulteration a non-paying business. "The fact that during the past year more than seven million pounds of food unfit for use have been seized and destroyed, and nearly three hundred persons engaged in the illegal traffic have been convicted and punished," said Dr. Martin, "shows that the health officials have not been idle during that time. But when the enormous quantity of food brought into the city each year, amounting to billions of pounds in weight, is considered, it can be seen readily that, to make such supervision faultless, the expense would be correspondingly enormous.

"There are, however," continued Dr. Martin, "a number of simple, inexpensive home tests, easily applied, and requiring only the expenditure of a little time, which would make every housewife her own food inspector, and which would do more than any official action could to lessen the evil effects of this nefarious traffic, which seeks profit at the expense of the good health of the community, and oftentimes at the cost of life itself." Among the home tests suggested by Dr. Martin for the detection of food unfit for use through adulteration, or from other causes, are the following:

The bright green color of pickles is often due to the presence of salts of copper in solution. To detect this crush a small piece of the pickle, place it in a cup with a bright, coarse needle, and, at the end of twenty minutes, if there was any copper in the pickle, the needle will be coated with a red film. This test can be made more interesting by placing the needle, after it is taken from the pickle, in a cup containing a teaspoonful of ammonia water. The latter will become bright blue in color if copper has been precipitated on the needle. Such pickles are harmful, and should be thrown away.

### THE BUTTER TEST.

Rancid or poor butter is easily detected by its taste or smell, but oleomargarine, which is a complete substitution of another substance in place of dairy butter, is difficult of detection even by experts. A simple test for suspected butter is to place some of it in a tin cup and heat it on the stove, at the same time stirring the substance with a fork. If it is oleomargarine, considerable spluttering and spitting will take place. On the other hand, genuine butter melts quickly with little or no noise.

In selecting canned food, always take the cans that have dents in them. Cans that are smooth and well rounded out are likely to be what are known as " swelled cans " which is caused by gas formed through fermentation. Dents in the can are proof of the absence of fermentation. When a can is opened the inner sides should be examined; if they are black and have evidently been acted upon by acids, the contents of the can should not be used. Canned corn, peas, or beans should never be sour. To detect sourness the litmus pencil, so called, is a useful household friend. One end of the pencil is blue and the other red. If too much acidity is suspected, put a little of the liquid on a piece of stout white paper, or if it is a solid moisten somewhat, and make a mark with the blue end of the pencil. If the substance is acid the blue mark will turn red at once. In a test for excessive alkali use the red end of the pencil, when the red mark will turn blue, if the substance is alkaline.

If a few grains of coffee are dropped into a quantity of cold water it will be found that, if the coffee is adulterated, more or less coloring matter will show in the water. Genuine coffee imparts no color to cold water. Another test is to take a needle and try to pick up grains of ground coffee; chickory and other adulterants are so soft that they are easily penetrated by the needle's point, while genuine coffee is hard and very difficult to be picked up in that way.

What is known as "lie" tea is often substituted in place of genuine tea. This "lie" tea is prepared by rolling up grains of sand with tea leaves already used, so as to imitate the weight and plumpness of genuine tea leaves. It can be detected by moistening the suspected tea and carefully opening the leaves with a needle, thereby disclosing the hidden grains of sand. Leaves of the oak, plum, peach, and sloe are also used as adulterants, and can be detected by comparison with leaves of genuine tea.

### FRESH FISH.

Freshly-caught fish have bright gills and clear, bead-like eyes; when fish become stale the gills assume a pinkish hue, and the eyes become whitish and opaque, and they remain so, no matter how often the fish dealer douches them with water to give them an appearance of freshness. A lobster that has been boiled after death can readily be detected by pulling the tail out straight. as the tail will then remain outstretched or curve inward again very slowly; whereas, if the lobster was boiled while alive the tail will spring quickly back into place.

The chief foreign ingredient in adulterated sugar, nowadays, is starch; to detect its presence a drop of tincture of iodine in a teaspoonful of water applied to the suspected sugar will cause a blue color to appear if starch or flour is mixed with the sugar. This test can be successfully used with any substance in which starch appears as an adulterant. To detect sand or other adulterant that is not soluble, dissolve a tablespoonful of sugar in a a bottle containing four or six ounces of water. Allow it to stand twenty-four hours, and the sediment, if any, which falls to the bottom of the bottle, will show the character of the adulterant. If there is sand the sediment will feel gritty to the touch; if there is carbonate of lime it will give off bubbles of gas when a few drops of vinegar are added and the sediment is moved.

A drop of artificially colored wine let fall into a tumbler of cold water will rapidly impart its color to the water. Genuine wine does not do this so readily. Another test is to moisten the fingers with wine and rub them quickly together. If the wine is artificially colored, the fingers will be stained; if genuine, they will not. The bitter taste of beer is often imitated in adulterations by an infusion of picric acid. To detect this, heat a cup of beer, made acid by adding a few drops of vinegar. While it is still warm immerse in it a few strands of white wool yarn, and if picric acid is present the wool will be dyed yellow.

### MILK TESTS.

A quick test of milk is to mix it well and then pour it from a glass. Pure, unadulterated milk will leave a thick coating on the inside of the glass, while watered or skim milk will run out cleanly. Good milk should contain 12 to 15 per cent. of cream, and the percentage of cream can be estimated in this way: Take a long, narrow bottle, with a capacity of six or eight ounces. Paste on the outside a strip of paper, half an inch wide, running from the neck to the bottom of the bottle; divide this strip by pencil marks into ten equal parts, fill the bottle to the five mark with milk, and then to the top of the paper with water at a heat which will just allow the hand to be inserted without causing pain, and to which as much soda has been added as would cover the end of a penknife. Shake well, to mix thoroughly, and place in an ice-box. In half an hour observe how much cream has risen, and measure this off on a piece of paper; double this distance, and then see what part of the total length of the paper strip on the bottle this is. If it is one-twentieth, the milk only contains 5 per cent of cream; if one-tenth, then it contains 10 per cent.; if one-fifth, then it contains twenty per cent., and so on.

A sure safeguard against ill effects from contaminated water, and especially useful in the country, or at summer resorts, is the addition of five drops of peroxide of hydrogen to the water a few minutes before drinking it. The liquid is obtainable at any drug store, is absolutely harmless, tasteless and colorless. Its addition to the water only breaks it up into active oxygen and water, the former killing whatever germs of disease may be present in the water. This method is equally efficacious and much more convenient than the ordinary way of boiling suspected water to destroy possible germs. If a few drops of solution of sugar are placed in a glass of suspected water, and the glass is covered and put in a warm place, the water, if it is contaminated, will become cloudy and deposit a sediment after a few hours.

*Indianapolis News*, July 9th, 1898.

